# Treatment Challenges and Controversies in the Management of Critically Ill Diabetic Ketoacidosis (DKA) Patients in Intensive Care Units

**DOI:** 10.7759/cureus.68785

**Published:** 2024-09-06

**Authors:** Bryan K Dunn, Hunter Coore, Navneeth Bongu, Kori L Brewer, Deepak Kumar, Anagha Malur, Hassan Alkhalisy

**Affiliations:** 1 Pulmonary and Critical Care Medicine, East Carolina University Brody School of Medicine, Greenville, USA; 2 Internal Medicine, East Carolina University Brody School of Medicine, Greenville, USA; 3 Pulmonary and Critical Care Medicine, Northeast Georgia Medical Center Gainesville, Gainesville, USA; 4 Emergency Medicine, East Carolina University Brody School of Medicine, Greenville, USA

**Keywords:** diabetic ketoacidosis (dka), cerebral edema and airway management, nutritional support, electrolyte management, insulin therapy, intravenous fluids, hyperosmolar hyperglycemic state (hhs)

## Abstract

This review discusses the challenges and controversies in the treatment of diabetic ketoacidosis (DKA) and hyperosmolar hyperglycemic state (HHS). Key areas include the selection of intravenous (IV) fluids, insulin therapy, strategies for preventing and monitoring cerebral edema (CE) by managing hyperglycemia overcorrection, electrolyte replacement, timing of nutrition, use of IV sodium bicarbonate, and airway management in critically ill DKA patients.

Isotonic normal saline remains the standard for initial fluid resuscitation, though balanced solutions have been shown to have faster DKA resolution. Current guidelines recommend using continuous IV insulin for DKA management after fluid status has been restored potassium levels have been achieved and subcutaneous (SQ) insulin is started only after the resolution of metabolic acidosis. In comparison, the British guidelines recommend using SQ insulin glargine along with continuous regular IV insulin, which has shown faster DKA resolution and shorter hospital stays compared to continuous IV insulin alone. Although rare, rapid overcorrection of hyperglycemia with fluids and insulin can lead to CE, seizures, and death. Clinicians should be aware of risk factors and preventive strategies for CE. DKA frequently involves multiple electrolyte abnormalities, such as hypokalemia, hypophosphatemia, and hypomagnesemia and regular monitoring is essential for DKA management. Early initiation of oral nutrition has been shown to reduce intensive care unit and overall hospital length of stay. For impending respiratory failure, Bilevel positive airway pressure is not recommended due to aspiration risks. Instead, intubation and mechanical ventilation, with monitoring and management of acid-base and fluid status, are recommended. The use of sodium bicarbonate is discouraged due to the potential for worsening ketosis, hypokalemia, and risk of CE. However, IV sodium bicarbonate can be considered if the serum pH falls below 6.9, or when serum pH is less than 7.2 and/or serum bicarbonate levels are below 10 mEq/L, pre-and post-intubation, to prevent metabolic acidosis and hemodynamic collapse that occurs from apnea during intubation.

Managing DKA and HHS in critically ill patients includes using balanced IV fluid solutions to restore volume status, followed by continuous IV insulin, early use of SQ glargine insulin, electrolyte replacement, and monitoring, CE preventive strategies by avoiding hyperglycemia overcorrection, early nutritional support, and appropriate airway management.

## Introduction and background

In the United States (US), diabetes mellitus (DM) affects 30 million people, and 1 in 10 individuals has diabetes [1]. Diabetic ketoacidosis (DKA) is a life-threatening medical emergency and is a direct result of a lack of insulin or insulin resistance, which leads to increased levels of counterregulatory hormones, such as glucagon, cortisol, and catecholamines, thereby causing hyperglycemia [2,3]. In the US, the average length of hospital stays for individuals with DKA is 3.4 days, and healthcare costs are 2.4 billion U.S dollars, highlighting the importance of the optimal management of DKA [1,3]. The overall mortality in adult patients with DKA admitted to the intensive care unit (ICU) ranges from 1 to 5%, and the mortality rate of patients with DKA not admitted to the ICU is approximately 1%. The mortality for patients with hyperosmolar hyperglycemic state (HHS) ranges from 10 to 20% [1,4,5,6,7]. Sepsis and cerebral edema (CE) are the most common causes of mortality in DKA patients and hyperglycemia overcorrection and sodium bicarbonate administration are risk factors [1,4,5,6]. The classical triad of DKA is hyperglycemia (glucose > 250 mg/dl), ketone production, and high anion-gap metabolic acidosis, which leads to severe metabolic abnormalities. DKA occurs more commonly in patients with type 1 diabetes than in type 2 diabetic patients. Patients with HHS have higher glucose levels (> 600), less acidosis (pH > 7.30), and lower serum ketones compared to DKA patients. DKA patients can have features of both DKA and HHS, as approximately 30% of patients with DKA have glucose levels > 600, resulting in an overlapping diagnosis of DKA and HHS [7]. Ketone body accumulation and ketosis development, mainly B-hydroxybutyrate and acetoacetate, occur due to a lack of insulin and excess glucagon DKA evolves over a 24-h period, with symptoms of polydipsia, polyuria, weight loss, confusion, abdominal pain, nausea, vomiting, and dehydration [8]. The overall management of DKA in critically ill patients includes correction of dehydration, electrolyte abnormalities, metabolic acidosis, and ketosis with intravenous fluid (IVF) replacement, insulin replacement, hemodynamic support, and airway management. In addition, any predisposing or precipitating factors, such as medical noncompliance, infections, stroke, myocardial infarction, and medications, should be identified and treated. Among the electrolyte disturbances encountered with DKA, potassium is the most important and abnormalities may lead to cardiac arrhythmias and even death.

The primary purpose of this article is to discuss and review the following challenges and controversies for the management of critically ill adult DKA patients in ICUs, IVF selection, insulin therapy, electrolyte replacement, risks and prevention strategies for hyperglycemia overcorrection, timing of initiating nutrition, sodium bicarbonate use and airway management. The American Diabetes Association (ADA) guidelines recommend using normal saline as the first line IVF for resuscitation, but a more balanced solution and less acidotic fluid (Lactated Ringer’s, PlasmaLyte, or Normosol) could be considered. Initiating continuous intravenous (IV) regular insulin drips after the volume status has been restored is the current standard of care, although the British guidelines recommend the administration of long-acting insulin glargine along with continuous IV insulin. Electrolyte imbalances of potassium, phosphate, and magnesium are common and can be life-threatening. Nutrition is typically held until acidosis has resolved, but there are no specific guidelines to support this practice. Because rapid overcorrection of hyperglycemia may lead to CE, seizures, and death, understanding the risk factors and implementing effective treatment strategies are crucial. The management of hypoxemia and airways is challenging, which makes the decision of when to intubate critically ill DKA patients particularly difficult. Traditional teaching is that DKA patients should not be intubated because it is nearly impossible to meet their high ventilatory demands without causing a hemodynamic collapse, but this method may not be supported by medical evidence. Sodium bicarbonate is usually contraindicated due to a risk of CE and potential worsening acidosis unless serum pH is < 7.0 or for life-threatening hyperkalemia. 

The pathophysiology of DKA and HHS is primarily due to either a deficiency in insulin production, which is more common in individuals with type 1 DM (leading to DKA), or a decrease in insulin effectiveness, typically observed in individuals with type 2 DM (leading to HHS). The elevation of counterregulatory hormones, such as cortisol, glucagon, catecholamines, and growth hormones, causes significant ketosis and hyperglycemia. This deficiency or ineffectiveness of insulin results in hyperglycemia, anion-gap metabolic acidosis, ketosis, and electrolyte disturbances, which causes fatty acids to be released from adipose, leading to ketone production of both acetoacetate and β-hydroxybutyrate [2,9].

DKA and HHS clinical presentations range from subtle symptoms to life-threatening complications due to a lack of insulin. The classic symptoms are polyuria, polydipsia, weight loss, dehydration, tachycardia, tachypnea, Kussmaul breathing, nausea, vomiting, and abdominal pain, which occurs due to osmotic diuresis from hyperglycemia. The diagnostic criteria for DKA and HHS are determined by glucose levels, acidosis degree, ketosis, osmolality, and neurological status (Table 1). 

**Table 1:** Classification of diabetic ketoacidosis (DKA) and hyperosmolar hyperglycemia state (HHS) Adapted from [2]

Serum Values	Mild DKA	Moderate DKA	Severe DKA	Hyperosmolar Hyperglycemia State (HHS)
Glucose (mg/dl)	> 250	> 250	> 250	> 600
pH	7.25-7.30	7.00-7.24	< 7.00	> 7.30
Bicarbonate (mEq/L)	15-18	10-15	< 10	> 18
Urine Ketones	Positive	Positive	Positive	Small or Absent
Serum Ketones	Positive	Positive	Positive	Small or absent
Beta-hydroxybutyrate (mOsm/kg)	3-4	4-8	> 8	< 0.6
Osmolality	Variable	Variable	Variable	> 320
Anion Gap	> 10	> 12	> 12	Variable
Neuro	Alert	Alert/Drowsy	Stupor/Coma	Stupor/Coma
Deficits				
Water (liters)	6 Liters	6 Liters	6 Liters	9 Liters
Water (ml/kg)	100	100	100	100-200
Sodium (mEq/kg)	7-10	7-10	7-10	5-13
Potassium (mEq/kg)	3-5	3-5	3-5	4-6
Phosphorous (mmol/kg)	5-7	5-7	5-7	3-7
Chloride (mEq/kg)	3-5	3-5	3-5	5-15

Significant abdominal pain is common in DKA/HHS patients and is associated with a degree of metabolic acidosis. Umpierrez and Freire investigated the incidence of abdominal pain in 189 patients with DKA and abdominal pain occurred in 46% of these patients, and there was a strong association between abdominal pain and the degree of metabolic acidosis [10]. In addition, abdominal pain was present in 86%, 66%, and 13% of patients with serum bicarbonate levels < 5 mmol/L, 5-10 mmol/L, and 15-18 mmol/L, respectively [7,10]. These findings suggest that there may be other causes of abdominal pain in DKA patients when serum bicarbonate levels are not significantly decreased. The most common complications of DKA are hyperglycemia, hypokalemia, acute hypoxemic respiratory failure, and aspiration pneumonia. Neurological manifestations of DKA range from mild confusion to coma to CE. Although rare, CE can occur, and it most commonly occurs in pediatric patients and HHS adult patients. Sodium bicarbonate is contraindicated unless the serum pH is < 6.9 due to a risk of worsening acidosis and CE. DKA causes stress on the pulmonary and cardiovascular systems, and it can lead to noncardiogenic pulmonary edema, pneumonia, acute respiratory distress syndrome, shock, and arrhythmias. Overall, key management strategies to prevent CE are to avoid aggressive lowering of glucose, aggressive IVF, or rapidly lowering serum osmolality.

## Review

**Figure 1:**
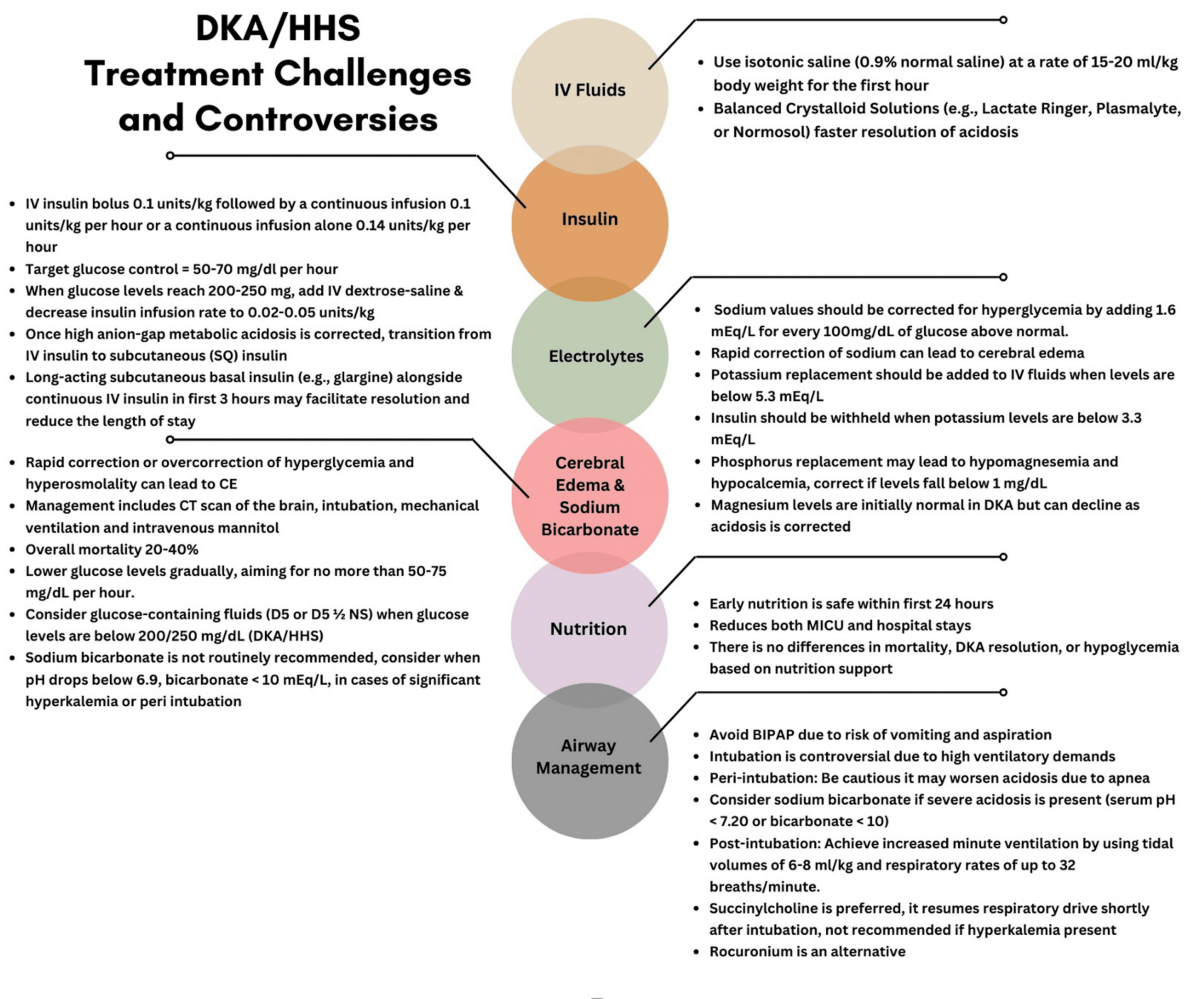
Summary of six DKA and HHS treatment challenges and controversies Image Credits: Deepak Kumar, MD and Bryan Dunn, MD DKA: Diabetic ketoacidosis; HHS: hyperosmolar hyperglycemic state; CE: cerebral edema; MICU: medical intensive care unit

Challenges and controversies for DKA management include IV fluid selection, insulin replacement, electrolyte replacement, cerebral edema, use of sodium bicarbonate, timing of nutrition, and airway management (Figure 1). DKA patients are initially hypovolemic and hyperosmolar, and IVF and electrolyte replacement are the cornerstone of treatment, followed by insulin therapy after the intravascular volume and tonicity have been restored. Current ADA guidelines recommend using isotonic saline (IS) (0.9% normal saline) at 15-20 ml/kg body weight for the first 1 h, followed by frequent assessment of volume status and replacement by monitoring hemodynamics, urine output, and physical exam [9]. When glucose levels are < 200 mg/dl, then dextrose-containing fluids should be initiated to allow the continuation of insulin therapy to treat ketosis. However, normal saline may cause hyperchloremic metabolic acidosis, which may worsen the underlying acidosis. Because a more balanced IVF may lead to faster resolution of acidosis (Table 2), a more balanced crystalloid solution (Lactated Ringer’s, PlasmaLyte, or Normosol, with pH ranging from 6.5 to 7.4) should be considered instead of normal saline [7,11-14].

**Table 2:** Composition of human plasma, normal saline, Ringer’s lactate solution, and PlasmoLyte/Normosol

Composition	Human Plasma	Normal Saline	Ringer’s Lactate	PlasmaLyte or Normosol	D5W and D10W
pH	7.4	5.0	6.5	7.4	4.4
Sodium (mEq/L)	135-145	154	130	140	0
Chloride (mEq/L)	94-111	154	109	98	0
Potassium (mEq/L)	4.5-5.0	0	4.0	5.0	0
Calcium (mEq/L)	2.2-2.6	0	3.0	0	0
Magnesium (mEq/L)	0.8-1.0	0	0	3.0	0
Buffer	Lactate 1-2	0	Lactate 28	Gluconate 23 Acetate	0
Osmolarity	285	308	274	295	252

Clinicians are hesitant to use Ringer’s lactate solution or balanced crystalloids (Normosol or Plasmolyte) due to the risk of hyperkalemia, but this tendency is not supported by evidence [7,12,14,15]. Approximately 98% of the total body potassium is intracellular, and normal saline can worsen acidosis, leading to a shift of potassium from the intracellular space (ICS) to the extracellular space (ECS). Dextrose should be added to IVFs (D5W or D5 1/2 NS) when serum glucose levels fall below 200-250 mg/dl to prevent hypoglycemia and risk of CE. The ongoing BRISK-ED pilot clinical trial by Yan et al. compares balanced crystalloids (Ringer’s lactate solution) to normal saline in adults with DKA in the Emergency Department to determine the ideal fluid selection for DKA patients [7]. Self et al. performed a subgroup analysis to investigate the clinical effects of balanced crystalloids versus saline in adults with DKA, and they suggested that a more balanced crystalloid solution can lead to a faster resolution of DKA compared to normal saline [11]. Catahay et al. performed a systematic review and meta-analysis, which demonstrated that the use of balanced electrolyte solution (BES) in DKA patients is associated with a faster resolution of acidosis compared to IS [16]. Current data suggest that a balanced crystalloid solution can lead to faster resolution of acidosis and DKA, suggesting its preference over normal saline [11-16]. 

In the US and based on current guidelines, IV insulin should be started after patients have received adequate IVF resuscitation and potassium levels have been achieved. Insulin corrects hyperglycemia and ketone production, and it inhibits glucagon and lipolysis [14]. Recommendations are to start IV insulin 0.1 units/kg bolus, followed by 0.1 units/kg/h or continuous insulin alone at 0.14 units/kg/h to decrease the glucose levels by 50-70 ml/dl/h. In most ICUs, the bolus is not usually given, and EndoTool or Glucomander software is used to assist with insulin dosing adjustments. When glucose levels are 200 or 250 mg/dl in patients with DKA or HHS, IV dextrose (D5) containing saline solution should be added, and insulin infusion should be decreased to 0.02-0.05 units/kg. In a prospective randomized controlled trial (RCT), Kitabchi et al. demonstrated no benefit from IV insulin bolus dosing compared to adequate continuous insulin infusions [15], and they concluded that a priming dose of insulin is not necessary if a sufficient continuous infusion rate (0.14 unit/kg/h) is provided for treating DKA. This conclusion challenges prior DKA protocols by suggesting an optimal infusion rate without the need for an initial bolus dose, and these findings suggest re-evaluating the necessity of an insulin priming dose to potentially simplify and streamline treatment protocols. Once the high anion-gap metabolic acidosis is corrected and patients can tolerate nutrition, patients can be transitioned from IV insulin drips to subcutaneous (SQ) insulin. Insulin dose requirements are calculated based on prior 24-hour requirements. The standard of care protocols in the US recommend utilizing IV continuous insulin during the acute phase of DKA treatment. SQ insulin administration is initiated once specific criteria are met, including resolution of acidosis, stabilization of glucose levels, and tolerance of oral intake. However, several publications support the concurrent use of long-acting SQ basal insulin (e.g., glargine) alongside continuous IV insulin, facilitating DKA resolution and reducing the length of stay (LOS) [16,17]. The 2023 Joint British Diabetes Society for Inpatient Care advocates for a regimen involving continuous IV insulin infusion at 0.1 units/kg in combination with long-acting SQ basal insulin. For patients already receiving basal insulin, their home dosage should be continued, while newly diagnosed individuals should start at 0.25 units/kg once daily [18]. In an RCT, Thammakosol and Sriphrapradang compared SQ insulin glargine (0.3 units/kg) within the first 3 h of DKA diagnosis plus standard IV insulin infusion to standard IV insulin infusion treatment and they found faster DKA resolution (mean time: 9.89 ± 3.81 h vs. 12.73 ± 5.37 h in control; p = 0.022) and shorter LOS in the early glargine group (median LOS: 4.75 days vs. 15.25 days in control; p = 0.024) [17]. This study reported a similar incidence of rebound hyperglycemia, all-cause mortality, hypoglycemia, and hypokalemia between groups. An early combination of insulin glargine with IV insulin infusion leads to faster DKA resolution and shorter LOS without increasing adverse outcomes, suggesting that this regimen could be considered [17].

The most common electrolyte abnormality in DKA is hyponatremia, which occurs from elevated plasma osmolality causing glucose levels to move water from the ICS to the ECS. Sodium levels are falsely elevated (termed pseudohyponatremia) with hyperglycemia and sodium values should be corrected by adding 1.6 mEq/L sodium for every 100 mg/ml of glucose above the normal glucose levels. Correcting sodium levels during DKA treatment will decrease the osmotic effect, allowing water to reenter the ICS. However, if sodium levels are corrected too rapidly, CE can occur.

Potassium levels are required before insulin is started. Both DKA and HHS patients can have 300-600 mMeq of potassium deficiency. Although most DKA and HHS patients will have normal or elevated potassium levels, 5% of these patients may have hypokalemia. Potassium losses occur due to glucose osmotic diuretics, gastrointestinal losses, and secondary hyperaldosteronisms. Potassium replacement should be added to IVFs when potassium levels are < 5.3 mEq/L and insulin therapy should be withheld when potassium levels are < 3.3 mEq/L.

IV insulin promotes the shifting of potassium to the ICS and increases the risk of arrythmias, muscle weakness, and pulseless electrical activity cardiac arrest. Patients often present with hyperkalemia, but 3-4% of cases can have hypokalemia [19,20]. In a recent case report, Grout et al. reported that a pediatric patient with DKA had resistant hypokalemia and subsequent ventricular tachycardia arrest when the patient’s potassium level was < 2.0 mEq/L while on insulin drip, providing evidence for withholding insulin therapy when potassium levels are < 3.3 mEq/L [19]. In addition, the case report by Murthy et al. on profound hypokalemia in a DKA patient showed the importance of IVF, potassium replacement, and airway management [20]. Although profound hypokalemia is uncommon with DKA, aggressive replacement is recommended, and insulin drip should be withheld when potassium levels are < 3.3 mEq/L [19,20]. If appropriate IV access is an issue, and the patient is critically ill, central venous access can be considered but is not without consequences. Gutierrez at el. reported that young pediatric patients with DKA have a higher incidence of deep vein thrombosis with femoral placed central line placement [21]. Phosphorus plays a crucial role in cellular metabolism and energy production. With DKA, phosphorus levels are often normal or initially elevated due to the release of intracellular phosphorus into the bloodstream in response to metabolic acidosis and insulin deficiency. However, despite this initial elevation, total body phosphorus stores can become depleted over time due to urinary losses caused by osmotic diuresis and renal dysfunction associated with DKA. Current guidelines suggest that phosphorus replacement should not be routinely administered unless serum phosphorus levels fall below 1 mg/dl, as supplementation may lead to potential complications, such as hypomagnesemia or hypocalcemia [22]. This cautious approach is essential because abrupt correction of phosphorus levels can exacerbate existing electrolyte imbalances and disrupt the delicate balance of mineral homeostasis. Magnesium is an essential electrolyte with several cellular functions, including enzyme activation, energy metabolism, and neuromuscular transmission. With DKA, serum magnesium levels are initially within the normal range. As acidosis is corrected, magnesium levels can decrease due to several factors, including increased renal excretion, redistribution of magnesium into cells, and urinary losses secondary to osmotic diuresis. One of the primary concerns is the potential impact on respiratory muscle function. Magnesium deficiency can lead to respiratory muscle weakness, impairing the ability of the diaphragm and intercostal muscles to generate adequate force for effective ventilation, which can result in respiratory compromise, leading to hypoventilation, hypoxemia, and respiratory failure. Hypomagnesemia can cause cardiac arrhythmias, particularly ventricular arrhythmias. Magnesium deficiency can also lead to hypokalemia and hypocalcemia.

The most feared complication from the treatment of hyperglycemia is CE, which occurs from rapid overcorrection of hyperglycemia and hyperosmolality, resulting in fluid shifting from the ECS to the ICS of the brain. CE occurs in 0.2-1.0% of pediatric patients, and it is much less common in the adult population [23-25]. Subclinical CE is much more common and may often go unrecognized [26]. Risk factors for the development of CE and subclinical CE include rapid infusion of insulin, rapid correction of hyperglycemia, significantly elevated glucose levels, younger age, new-onset diabetes, severe acidosis, uremia, first episode of DKA, slower correction of hyponatremia, sodium bicarbonate administration, and production of osmotically active ions in the brain [27-29]. A case report by Varela et al. reviewed a 31-year-old male with new-onset diabetes who presented with DKA and HHS, leading to CE and neurologic deficits from rapid overcorrection of glucose > 2000 mg/dl. The term idiogenic osmoles are osmotic active solutes produced by the central nervous cells in response to elevated omsoles from hyperglycemia later during DKA [24]. These ions are produced in the brain and are slow to clear with DKA, which may lead to fluid shifting from the ECS to the ICS, causing hypertonicity and brain swelling, ultimately leading to CE [28,29]. Fluid can shift from the ECS to the ICS, which leads to a rapid correction of hyperglycemia and hyperosmolarity, resulting in CE. Sodium levels increase in response to this correction, which may help prevent a rapid decrease in serum osmole levels.

Current guidelines recommend that patients with DKA be adequately resuscitated followed by insulin administration. However, there are no current guidelines for the rate at which glucose levels should be lowered in adults. Pediatric guidelines recommend that IVFs be administered at a rate of 15-20 ml/kg/h over the first several hours (1.05-1.4 L/h in 70 kg patient) and that glucose levels should be lowered no more than 50-75 mg/dl/h (300-600 mg/dl in first 6 h). Glucose-containing fluids (D5 in water or D5 1/2 normal saline) should be administered when glucose levels are < 200/250 mg/dl (DKA/HHS patients), with the goal of lowering serum osmolality < 3 mmol/kg/h and no more than 10 mmol/24 h (275-295 mmol/kg is normal) [25]. Frequent neurological checks are recommended during the first 12 h of treatment for DKA to monitor for CE. Symptoms of CE include acute encephalopathy, seizures, bradycardia, irregular respirations, and hypertension. If CE is suspected or occurs, then intubation, mechanical ventilation, IV mannitol, and a CT scan of brain should be considered. The overall mortality of DKA with CE is 20-40%, which is greater than that of DKA without CE (1%) [25].

The administration of sodium bicarbonate (NaHCO3, 8.4%) as a replacement therapy for metabolic acidosis in patients with DKA is not routinely recommended. However, the use of sodium bicarbonate may be warranted under specific circumstances. Sodium bicarbonate is typically indicated when arterial blood gas (ABG) pH levels drop below 6.9 or in cases of significant hyperkalemia (greater than 6.4 mEq/L). Additionally, sodium bicarbonate can be considered peri-intubation if the ABG pH level is < 7.20 or if the sodium bicarbonate level is <10 mEq/L. Despite these indications, there have not been any RCTs evaluating the efficacy and safety of sodium bicarbonate administration in DKA patients. However, existing observational data raise concerns regarding potential adverse effects [30]. Sodium bicarbonate administration has been associated with various detrimental outcomes, including reduced ventilatory drive, elevated levels of partial pressure of carbon dioxide (PaCO2), exacerbation of overall acidosis, exacerbation of overall of ketosis, increased lactate acid production, and hypokalemia. Furthermore, sodium bicarbonate administration may lead to a leftward shift of the oxyhemoglobin dissociation curve, potentially impairing oxygen delivery to tissues [30]. 

The current expert opinion suggests considering the use of sodium bicarbonate in cases of severe DKA, particularly before intubation. This recommendation aims to minimize the risk from hypercapnia, resulting from apnea during rapid sequence intubation (RSI) [30]. However, the decision to administer sodium bicarbonate should be made on a case-by-case basis, weighing potential benefits against the associated risks and considering the patient's overall clinical condition and individual factors. RCTs specifically evaluating the use of sodium bicarbonate in this patient population would be helpful in determining the efficacy and safety profile of sodium bicarbonate.

It is currently recommended that enteral nutrition in DKA patients should not be initiated until the acidosis and anion gap have been resolved, but there are no specific guidelines to support this recommendation. Lipatov et al. addressed this question in a retrospective cohort study comparing early (< 24 h) versus late oral nutrition (> 24 h) in DKA patients in the ICU [31]. They reported that initiating nutrition early is safe and reduces ICU and hospital stays, and they showed no differences in mortality, DKA resolution, or hypoglycemia between the early and late nutrition groups. Additionally, these researchers observed a significant decrease in hypokalemia and hypophosphatemia in the early nutrition group. However, this study was underpowered, and further studies would be beneficial.

DKA and HHS can result in acute encephalopathy, stupor, and coma, which can lead to aspiration, hypoxemia, hypercapnia, respiratory failure, and cardiac arrest. Metabolic acidosis, electrolyte disturbances, and dehydration affect airway tone and respiratory status, potentially leading to airway compromise, aspiration pneumonia, and the need for intubation and mechanical ventilation. Kussmaul breathing, also known as metabolic or acidotic breathing, is a hallmark manifestation of DKA, and it occurs as a compensatory response to severe metabolic acidosis. Treatment strategies vary from supplemental oxygen (nasal cannula, high-flow nasal cannula, or non-rebreather) to more invasive interventions with intubation and mechanical ventilation. Bilevel positive airway pressure (BIPAP) should be avoided due to the risk of vomiting and aspiration. The management of both hypoxemia and hypercapnia in critically ill DKA patients in the ICU is challenging, and the timing of intubation in these patients is controversial. Traditionally, intubating DKA patients is discouraged, as matching their high ventilatory demands is nearly impossible. Following intubation and post-vent management, the goal is to achieve a higher minute ventilation with tidal volumes (6-8 ml/kg) and respiratory rates (up to 32) to prevent exacerbating existing high anion-gap metabolic acidosis with post-intubation respiratory acidosis. Clinicians should obtain frequent ABG samples post-intubation (every 15-30 minutes) and consider end-tidal CO2 (ETCO2) to ensure adequate minute ventilation (6 of 8 liters/min).

Airway management involves the use of RSI to increase first-chance success [32]. According to multiple studies, succinylcholine can lead to a potassium increase of 0.5 mEq/L, although is usually clinically insignificant and can be used if potassium levels are not significantly elevated (< 5.5 mEq/L) [32,33]. Zink et al. noted a lack of hyperkalemic response in patients receiving succinylcholine, with potassium levels increasing in 46 patients, decreasing in 46 patients, and remaining unchanged in eight patients [34]. Based on current recommendations, succinylcholine, and rocuronium can be safely used for RSI for intubations in DKA patients. The short-acting depolarizing neuromuscular blocker, succinylcholine, may allow patients to resume their own respiratory drive shortly after intubation, maintaining pre-intubation minute ventilation and preventing complications of worsening acidosis and shock. Succinylcholine should not be used if risk factors for hyperkalemia are present or if potassium levels exceed 5.5 mEq/L; in such cases, rocuronium should be utilized [33].

## Conclusions

DKA clinical manifestations occur from a lack of insulin, which causes hyperglycemia, metabolic acidosis, ketosis, and electrolyte abnormalities. If left untreated, DKA can progress to cardiovascular and pulmonary arrest. This article reviews six challenges and controversies in DKA management. Fluid replacement is a critical first-line therapy for DKA management. While current guidelines recommend isotonic saline (0.9% normal saline) for fluid resuscitation, recent data suggest that using a more BES may lead to faster resolution of DKA and could be considered. Continuous IV insulin should be started after restoring volume status and potassium levels have resulted. Insulin is continued until acidosis resolves when IV insulin can be transitioned to SQ insulin. British guidelines recommend introducing SQ insulin glargine within the first three hours of DKA diagnosis alongside continuous IV insulin therapy. This approach has been shown to achieve faster resolution of DKA and reduced length of hospital stay, with no increase in adverse outcomes. Rapid overcorrection of hyperglycemia with insulin can result in CE, seizures, and even death. IV sodium bicarbonate not routinely be used and reserved for patients with a serum pH <6.9, significant hyperkalemia, or during peri-intubation when pH is < 7.2 or sodium bicarbonate is < 10. Early initiation of nutrition within 24 hours has been found to be safe and reduces both MICU and overall hospital length of stays. For impending respiratory failure, BIPAP should not be used due to aspiration risks and intubation is recommended. By understanding these challenges and controversies in DKA management, clinicians can improve and optimize patient outcomes for critically ill DKA patients.
